# Metastable Ni(I)-TiO_2–*x*_ Photocatalysts: Self-Amplifying H_2_ Evolution from Plain Water without Noble Metal Co-Catalyst and Sacrificial Agent

**DOI:** 10.1021/jacs.3c08199

**Published:** 2023-11-20

**Authors:** Marco Altomare, Shanshan Qin, Viktoriia A. Saveleva, Zdenek Badura, Ondrej Tomanec, Anca Mazare, Giorgio Zoppellaro, Alberto Vertova, Angelo Taglietti, Alessandro Minguzzi, Paolo Ghigna, Patrik Schmuki

**Affiliations:** † PhotoCatalytic Synthesis PCS Group, MESA+ Institute for Nanotechnology, University of Twente, P.O. Box 217, Enschede 7500 AE, The Netherlands; ‡ Department Materials Science WW-4, LKO, Friedrich-Alexander-University of Erlangen-Nuremberg (FAU), Erlangen 91058, Germany; § ESRF, The European Synchrotron, 71 Avenue des Martyrs, CS40220, Grenoble Cedex 9 38043, France; ∥ Regional Centre of Advanced Technologies and Materials, Czech Advanced Technology and Research Institute, Palacký University Olomouc, Křížkovského 511/8, Olomouc 779 00, Czech Republic; ⊥ Nanotechnology Centre, VŠB − Technical University of Ostrava, 17. listopadu 2172/15, Ostrava-Poruba 708 00, Czech Republic; # Dipartimento di Chimica, Università degli Studi di Milano, Via Golgi 19, Milan 20133, Italy; ∇ Dipartimento di Chimica, Università degli Studi di Pavia, Viale Taramelli 13, Pavia 27100, Italy

## Abstract

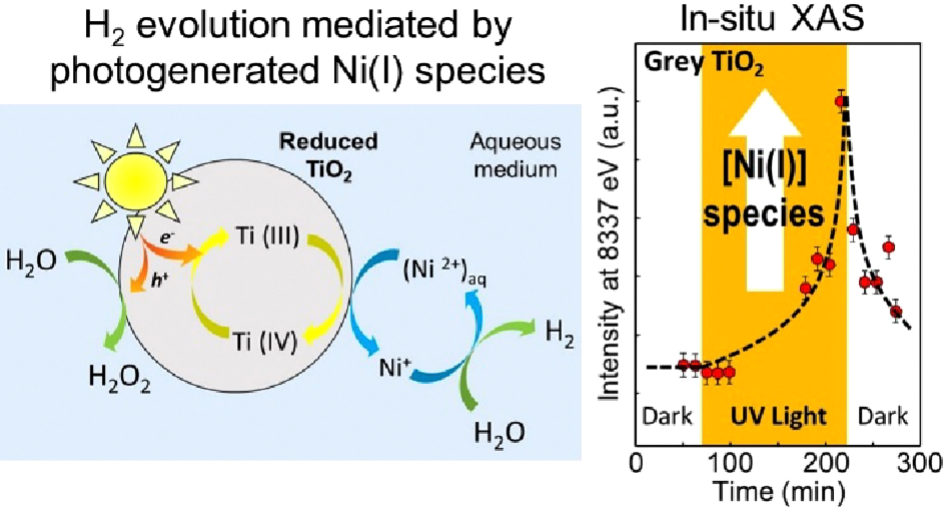

Decoration of semiconductor photocatalysts with cocatalysts is generally done by a step-by-step assembly process. Here, we describe the self-assembling and self-activating nature of a photocatalytic system that forms under illumination of reduced anatase TiO_2_ nanoparticles in an aqueous Ni^2+^ solution. UV illumination creates *in situ* a Ni^+^/TiO_2_/Ti^3+^ photocatalyst that self-activates and, over time, produces H_2_ at a higher rate. *In situ* X-ray absorption spectroscopy and electron paramagnetic resonance spectroscopy show that key to self-assembly and self-activation is the light-induced formation of defects in the semiconductor, which enables the formation of monovalent nickel (Ni^+^) surface states. Metallic nickel states, i.e., Ni^0^, do not form under the dark (resting state) or under illumination (active state). Once the catalyst is assembled, the Ni^+^ surface states act as electron relay for electron transfer to form H_2_ from water, in the absence of sacrificial species or noble metal cocatalysts.

## Introduction

In materials science, phenomena of self-assembly can be observed on a wide range of length scales, and many examples are relevant to create specific functional materials.^1^ Some important photoinduced processes are based on self-aggregation, -formation, and -organization.^2^ Such self-assembly processes that occur by “simmering in one pot for sufficient time” represent a stark contrast to the general synthesis strategies used in a chemical or materials science laboratory. In classic synthesis approaches, complex functionality is achieved by a careful step-by-step procedure that targets an optimization of each step. For example, photocatalysts are typically designed from a semiconductor that allows for light harvesting and charge carrier generation, while cocatalysts are decorated onto the semiconductor to overcome the intrinsic kinetic hindrance of many important photocatalytic reactions. In fact, from a purely thermodynamic standpoint, the position of band edges in TiO_2_ can enable the reaction of H_2_O into H_2_, O_2_, and •OH radicals as well as H_2_O_2_.^3−6^ Nonetheless, even with the thermodynamic advantages for water splitting into H_2_ and O_2_, unaltered (stoichiometric) phases of TiO_2_ exhibit unsatisfactory rates of H_2_ evolution and undetected O_2_ evolution from pure water, as well documented in the literature.^7,8^ The observation that O_2_ generation is less than stoichiometric or entirely absent can be ascribed not only to the sluggish kinetics of the oxygen evolution reaction (OER) in the absence of any catalysts but also to the fact that photoreduced TiO_2_ tends to strongly adsorb oxygen as O_2_^–^ or as O_2_^2–^.^9^

More generally, for many semiconductors without cocatalysts, O_2_ and H_2_ evolution reactions are kinetically hampered.^10−12^ To overcome this limitation, noble metals such as Pt, Pd, Rh, among others, are decorated in the form of cocatalyst nanoparticles on the semiconductor surface for H_2_ evolution.^13−16^ To promote hole-transfer toward O_2_ evolution, catalysts such as IrO_2_ or RuO_2_^17,18^ are typically used. Electron and hole transfer processes can also be facilitated using sacrificial agents.^19,20^

In recent years, research on cocatalysts increasingly focused on replacing “costly” noble metals with more economic alternatives, such as sulfides or phosphides^21−24^ of various transition metals, or their alloys.^25−29^ Among the latter, particularly Ni and its alloys have attracted wide interest, particularly for the surface modification of TiO_2_ and SrTiO_3_ photocatalysts.^30−34^ The CB and VB edges of TiO_2_ are positioned at −0.5 and +2.5 eV, respectively,^4^ while the CB and VB edges of SrTiO_3_ lay at about −0.8 and +2.5 eV, respectively^35^ (vs SHE, pH 7). The electrochemical potential of the Ni^2+^/Ni redox couple is −0.64 V (vs SHE, pH 7). In this framework, Ni has been found to be bifunctional, i.e., it can serve in the metallic form as cocatalyst for H_2_ evolution and in the oxidized form (oxyhydroxide) as cocatalyst for O_2_ generation.^35^ Some reports suggest that Ni oxide or hydroxide placed on various semiconductors can be reduced to metallic Ni by photogenerated conduction band (CB) electrons.^35^ However, there are controversial results on the photocatalytic reduction of Ni^2+^ species from solution to metallic Ni on a photocatalyst surface. Reduction of Ni^2+^ to metallic Ni has been reported for cases where holes in the illuminated semiconductor are rapidly consumed by a hole scavenger (e.g., methanol, ethanol, etc.).^36,37^ We confirmed this in previous work using *in situ* X-ray absorption spectroscopy (XAS) to study Ni-, Cu-, and NiCu-modified TiO_2_.^38^ For Ni-modified SrTiO_3_, in pure water, it has been reported that photocarrier transfer may lead to disproportionation reactions forming both Ni and NiO_*x*_ sites^35^ or Ni/NiO_*x*_ core–shell NPs.^39^ Such NiO_*x*_ compounds or Ni/NiO_*x*_ core–shell nanostructures are then able to form a NiO_*x*_ cocatalyst for water oxidation to O_2_.

In contrast to SrTiO_3_, and some other complex semiconductors, for titanium dioxide (TiO_2_), these types of reactions are not observed in plain water. That is, to achieve facile electron and hole transfer from TiO_2_ to water, a noble metal cocatalyst as electron transfer mediator and an efficient hole-capturing species (such as methanol) are typically required.^19,20^ However, in such cases, holes react with the sacrificial agent, and methanol photoreforming replaces the water oxidation reaction.^20^ In plain water, on TiO_2_, the hole transfer reaction to water and the formation of O_2_ are strongly hampered.^20,40^ Interestingly, some works reported that on Pt-decorated titania surfaces, a two-hole transfer to water is kinetically favored, leading to H_2_O_2_ rather than O_2_ as reaction product.^41−44^ However, also this two-hole pathway to H_2_O_2_ is extremely slow on a pristine TiO_2_ surface, and thus the hole transfer reaction represents the real bottleneck in TiO_2_ photocatalytic water splitting. In a previous study, we reported that so-called *gray* TiO_2_ can provide long-lived holes that enable this two-hole pathway to H_2_O_2_.^4^*Gray* anatase is a form of reduced anatase TiO_2_, produced by thermal hydrogenation at 500 °C, possessing defective surface structure that can promote electron and hole transfer.^45^ As a result, *gray* titania decorated with metallic Ni particles was found to be an effective photocatalyst for the splitting of plain water into H_2_ and H_2_O_2_.^4^

Here, we demonstrate a more remarkable use of *gray* anatase, that is, the *in situ* formation of a metastable photocatalyst for H_2_ evolution from an aqueous Ni^2+^ solution. This takes place in the absence of sacrificial species or noble metal cocatalysts and without the formation of metallic Ni nanoparticles by photoreduction on the surface of *gray* TiO_2_. *In situ* XAS and electron paramagnetic resonance (EPR) spectroscopy show light-induced formation of defects in the semiconductor combined with the formation of surface trapped monovalent Ni^+^ states. We propose that such Ni^+^ states act as a relay for electron transfer to form H_2_ from water. This property is distinct to TiO_2_ powders hydrogenated under optimal conditions, as both nonreduced *white* and over-reduced *black* titania show negligible H_2_ evolution under comparable photocatalytic conditions.

## Results and Discussion

**Figure 1:**
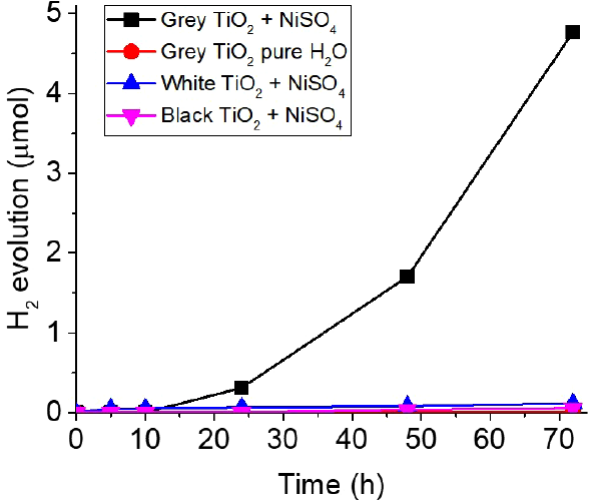
H_2_ evolution performance: comparison of *gray* TiO_2_ nanoparticles with *white* and *black* TiO_2_ nanoparticles in 0.4 mM NiSO_4_·6H_2_O aqueous solution under illumination.

In a simple gastight photoreactor (quartz tube), mildly reduced anatase titania nanopowders (*gray* titania^45^) are dispersed in an aqueous 0.4 mM NiSO_4_ solution and illuminated with UV light (UV LED, 365 nm, 100 mW cm^–2^). Under these experimental conditions, in a few hours, an increasingly effective photocatalytic system self-assembles and a metastable state is established that produces H_2_ from water. This self-activation process is illustrated in Figure 1 (see the Supplementary Information for additional experimental details and Figure S1 for further characterization of *gray* TiO_2_). After switching on UV illumination, during the first few hours of illumination, only negligible amounts of H_2_ can be detected (such amounts are in the range of or below the detection limit of the gas chromatograph, GC). Then, the H_2_ evolution becomes evident, and after 10 h of illumination it keeps accelerating, e.g., until 72 h of illumination (at this time the UV light was turned off and the experiment was stopped). Interestingly, these self-activation and amplification phenomena take place in the absence of any sacrificial agent. We observed a similar effect in previous work where we hypothesized the formation of a monovalent nickel cocatalyst mediator.^46^ Here, we prove our hypothesis by *in situ* XAS and EPR spectroscopy.

This effect is unique to *gray* anatase and cannot be observed for *white* TiO_2_ (untreated anatase) or *black* TiO_2_ (Figure 1). *Black* TiO_2_ is a form of over-reduced (highly defective) titania produced by hydrogenation under harsh conditions (700 °C in this case).^47−50^*Black* and *gray* TiO_2_ differ in the reduction treatment used to produce the materials from *white* (untreated) anatase. Consequently, the three forms of titania herein studied exhibit different density and nature of oxygen vacancies (Ti^3+^-O_V_) and, thus, different electronic properties.^45^ We characterized these materials in view of their electronic and defect structure in previous work.^4,45,50−56^ Unlike low temperature treatments (e.g., 500 °C) that maintain anatase as the only crystalline phase, hydrogenation at 700 °C (to form the over-reduced black TiO_2_) results in a mixed anatase–rutile composition.^45^ Among different “shades” of *gray* titania, i.e., thermally treated in H_2_ at different reduction temperatures (300–700 °C), the powder treated at 500 °C for 1 h (*gray* TiO_2_) shows the highest photocatalytic activity (shown in our previous work^46^).

Figure 1 shows that an incubation period is observed until H_2_ evolution is detected in significant amounts. It is known that illumination of anatase in aqueous methanolic solutions^57^ forms Ti^3+^ sites that are catalytically active for the generation of H_2_.^58^ Differently, in the present work, we show how UV light can form Ti^3+^ sites that generate H_2_ from plain water, i.e., in the absence of any hole scavenger, due to the presence of Ni^2+^ and via the formation of a metastable Ni^+^/TiO_2_/Ti^3+^ photocatalyst (discussed below). The key role of Ni ions is evident from data in Figure 1, i.e., when comparing the H_2_ evolution measured in an aqueous NiSO_4_ solution vs Ni^2+^ free solution. Additional data on the effect of pH, temperature, nickel salt precursor, and illumination wavelength and power density on the photocatalytic H_2_ evolution rate can be found in Figure S2.

**Figure 2:**
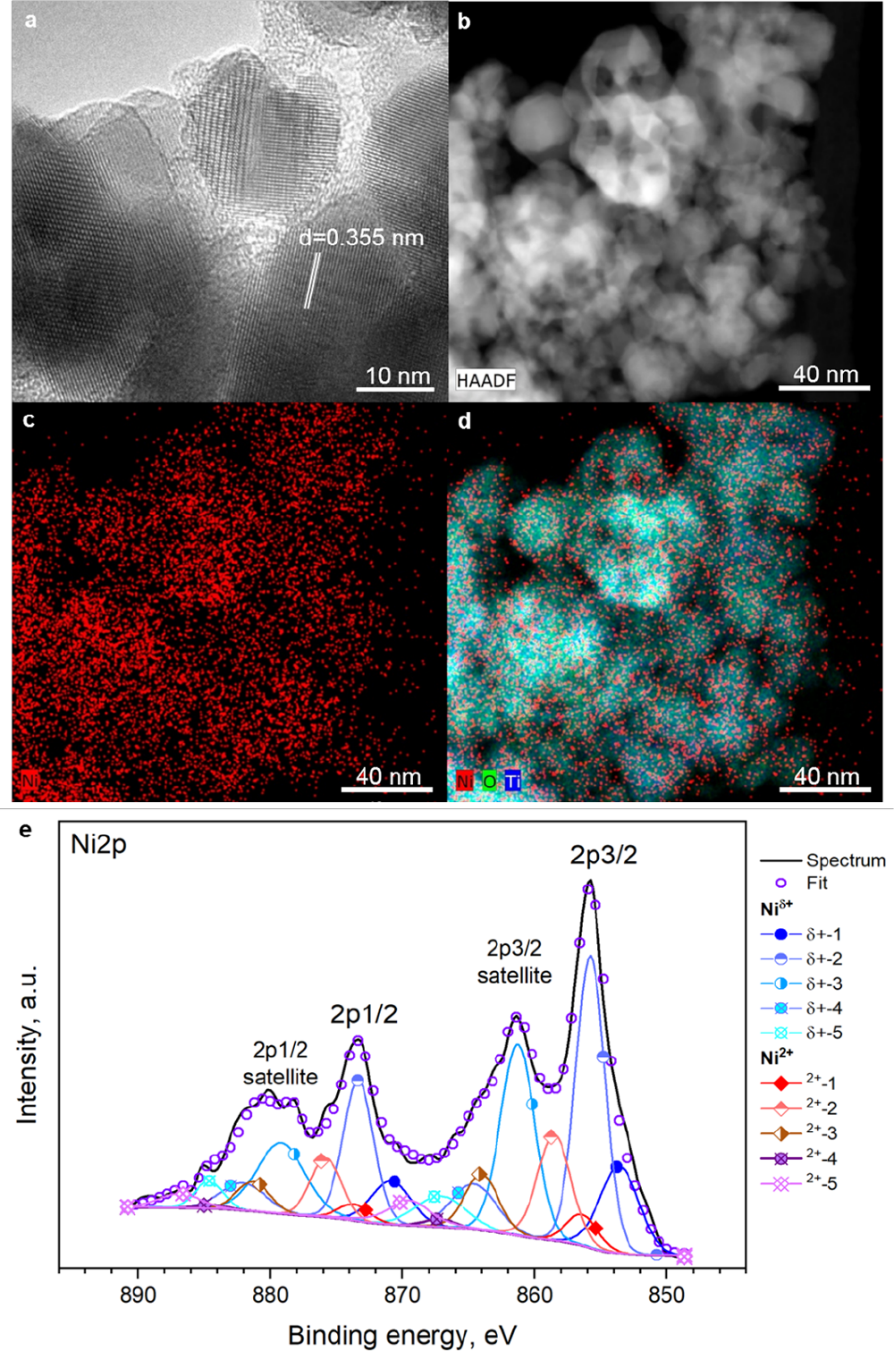
(a–d) *Gray* TiO_2_ nanoparticles after illumination for 10 days in a 0.4 mM NiSO_4_·6H_2_O aqueous solution: (a) High-resolution TEM image. (b) High-angle annular dark-field imaging (HAADF). TEM–EDS elemental mappings of (c) Ni and (d) Ni, O, and Ti. (e) Peak fitting of the high resolution XPS spectrum in the Ni 2p region for the sputtered anatase TiO_2_ layer (hydrogenated at 500 °C) illuminated with a 365 nm LED (100 mW cm^–2^) for 24 h in a 0.4 mM NiSO_4_·6H_2_O aqueous solution.

A first assumption may be that photocatalytic reduction of Ni^2+^ to Ni° would occur under UV irradiation. In this case, over time, the titania would be decorated with Ni° nanoparticles that act as cocatalyst for H_2_ evolution. However, we find that after illumination (even up to 10 days), no morphological trace of particle deposition can be observed on *gray* titania, neither by extensive investigations by scanning electron microscopy (SEM, Figure S3) nor by high-resolution transmission electron microscopy (HR-TEM, Figure 2a,b). Nevertheless, the presence of Ni species can be clearly detected by TEM–energy dispersive spectroscopy (TEM–EDS, Figure 2c,d), which indicates that Ni is distributed uniformly on the nanopowder. The absence of particle deposition along with the evidence of a homogeneous Ni distribution hints to atomically dispersed Ni species. Similar species are commonly encountered in single atom catalysis.^59−61^

For powder samples, the amount of Ni is below the detection limit of X-ray photoelectron spectroscopy (XPS). However, if compact, polycrystalline anatase layers produced by magnetron sputtering (on SiO_2_/Si) and subsequently reduced (hydrogenation at 500 °C, 1 h) are illuminated (24 h, UV light) in a 0.4 mM NiSO_4_·6H_2_O aqueous solution, XPS can reveal the presence of Ni species (Figure 2e). These layers show an onset of H_2_ evolution in analogy to the powder (Figure S4), and no morphological trace of photodeposited Ni particles can be observed (Figure S5). The XPS data reveal a Ni surface content of ca. 0.5 at. %, and a Ni:Ti ratio of ca. 0.04:1, indicating a low loading of Ni species (see Table S1).

The fitting of the Ni 2p XPS peak suggests the presence of Ni in various oxidations states. First, the Ni 2p consists of the Ni 2p_3/2_ and Ni 2p_1/2_ peaks at 855.9 and 873.5 eV, respectively, and with the corresponding satellite peaks at 861.3 and 879.9 eV (consistent with literature data).^62^ To evaluate the chemical states of Ni, peak fitting of the Ni 2p spectrum in Figure 2e was performed. For the fitting, we used empirical fits obtained from parameters derived from standard samples typically found in literature.^63−66^ The detected species include Ni^δ+^, typical of surface-coordinated Ni states, and Ni^2+^. Consistent with SEM and TEM, no metallic Ni was detected (typical binding energy of 852.6 eV). The binding energy, full width half-maximum and percentage of the peak area of the fitted peaks comprising the 2p_3/2_ peak and corresponding satellite are listed in Table S2 in the Supporting Information and are consistent with literature data.^62−64^ The fitted spectrum with an error below 0.5% includes the 2p_3/2_, 2p_1/3_ and satellite peaks for Ni^δ+^ (72.4%) and Ni^2+^ (27.6%). However, the *ex situ* XPS data may be affected by exposure of the sample to air due to sample removal from the electrolyte and transfer to the XPS setup. In general, this issue may affect *ex situ* characterization results.

**Figure 3:**
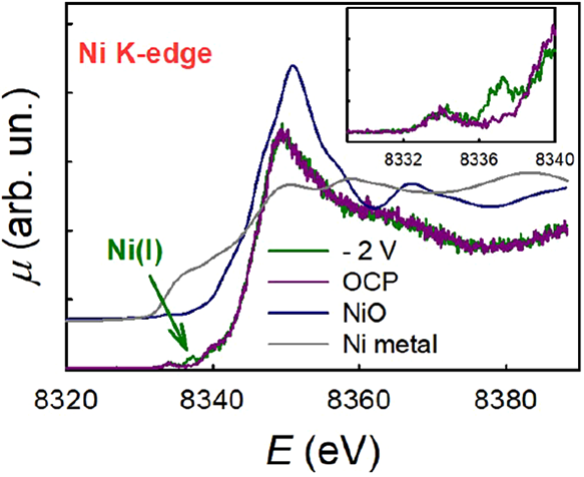
XANES spectra of the [Ni(cyclam)]^2+^ complex at the OCP and −2 V. Ni and NiO spectra are shown as a reference for Ni(0) and Ni(II) oxidation states, shifted along the *y* axis for better clarity. Inset: pre-edge region for the [Ni(cyclam)]^2+^ complex at the OCP and −2 V.

Therefore, to clarify the self-activation mechanism and the role of Ni in the photocatalytic system, we performed *in situ* XAS experiments. Experimental details are provided in the Supporting Information. In a preliminary experiment, we studied the electrochemical reduction of a Ni(II) complex [Ni(cyclam)]^2+^ in an acetonitrile-based electrolyte to obtain a reference spectrum for *in situ* formed Ni(I) species; [Ni(cyclam)]^2+^ is in fact known to undergo reduction to formal Ni(I) species.^67,68^ Experimental details on the synthesis and characterization of the Ni(II) complex [Ni(cyclam)]^2+^ are provided in the SI. The X-ray absorption near-edge structure (XANES) spectrum of the complex at the Ni K-edge is shown in Figure 3.

We measured the spectra of the complex under open circuit potential (OCP) conditions. As a reference for Ni(II), we use here a Ni K-edge XANES spectrum of NiO. The close correspondence in energy of the edge position and the overall similarity of the spectral shapes clearly demonstrate that the [Ni(cyclam)]^2+^ complex features divalent nickel centers. We then polarized the working electrode (a transparent conductive oxide-coated glass slide, FTO) at −2 V (vs Ag pseudoreference electrode), since preliminary voltametric experiments showed a one-electron reduction peak to Ni(I) at about −1.8 V^69^ (see CV in Figure S6). In the Ni K-edge spectrum, a new structure at ca. 8337.1 eV appears (green arrow in Figure 3, highlighted in the inset). We can refer to this feature as the signature of Ni(I). In addition, the spectrum at −2 V clearly indicates that Ni^0^, i.e., metallic nickel, does not form.

**Figure 4:**
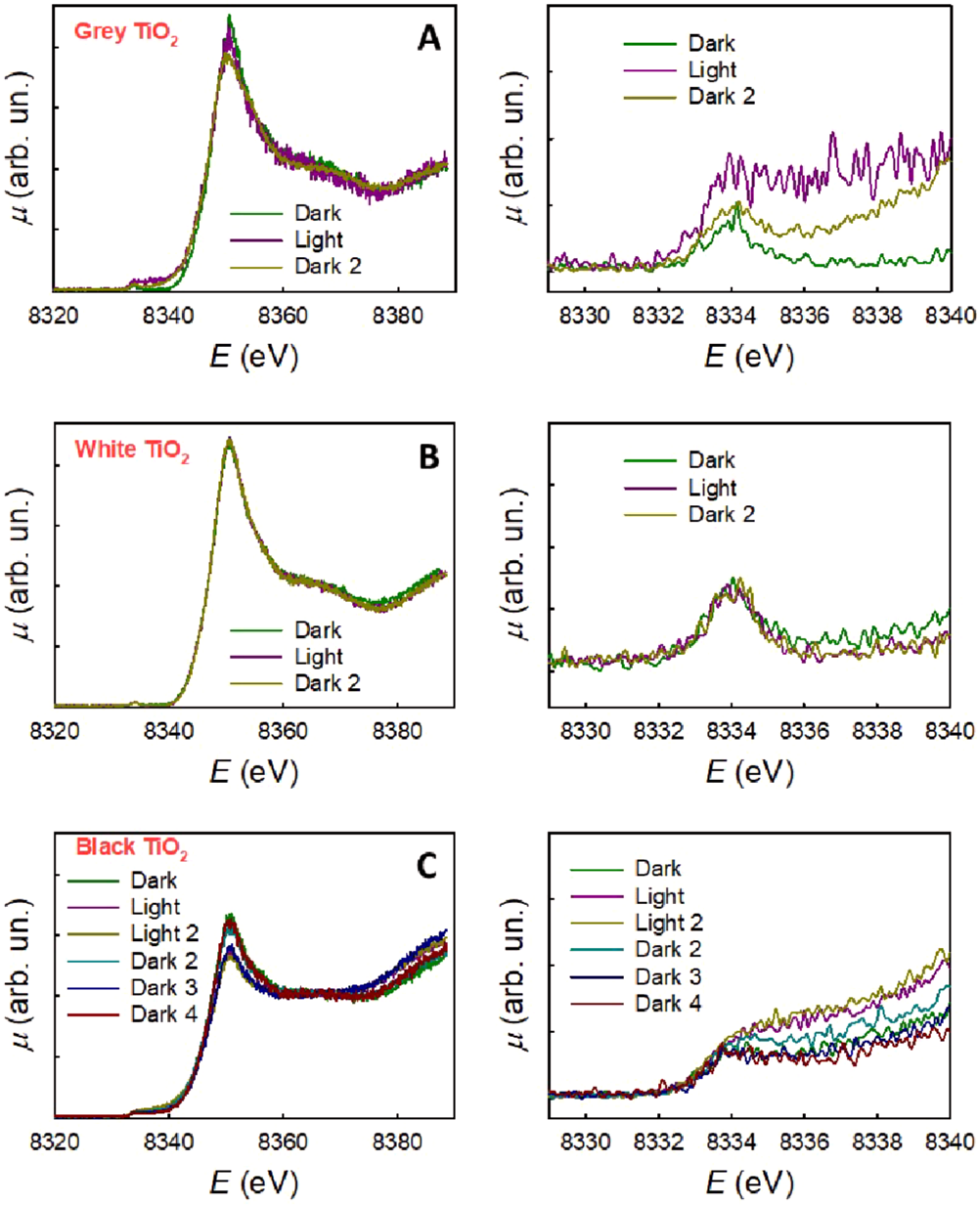
XANES spectra of *gray* (a), *white* (b), *and black* (c) TiO_2_ in a 0.4 mM solution of NiSO_4_ under intermittent UV light illumination. Right panels: magnified pre-edge regions.

We then performed *in situ* XAS experiments studying the different TiO_2_ photocatalysts in aqueous suspensions in the presence of NiSO_4_, both under dark conditions and under UV light illumination (i.e., under photocatalytic H_2_ evolution conditions). A picture of the setup along with experimental details and can be found in Methods and Figure S7, respectively. The XANES spectra at the Ni K-edge of *gray* TiO_2_ (in a 0.4 mM solution of NiSO_4_) under different conditions are shown in Figure 4a. In the dark, the spectrum resembles that of Ni(II) hydrated ions. Figure S8 shows the Ni K-edge XANES spectrum of a 0.4 mM aqueous solution of NiSO_4_, compared to the spectra of the same solution in the presence of black, gray, and white TiO_2_, all under dark conditions. The close similarity of the absorption edge position for all of the spectra confirms that the oxidation state is Ni(II) in every case. Minor differences are visible when the solution is put in contact with the different TiO_2_ powders, which may be indicative of the formation of different Ni(II) species adsorbed onto the TiO_2_ nanoparticles.

Upon illumination, a new broad feature appears at ca. 8336–8338 eV, and this is indicative of the formation of Ni(I). We note that this structure in the presence of TiO_2_ is broader when compared to the signal obtained by electrochemical reduction of the [Ni(cyclam)]^2+^ complex (Figure S9). This may be related to the fact that the Ni(I) sites are adsorbed on the TiO_2_ nanoparticle surface and therefore are expected to experience significant local disorder. Note that no Ni^0^, i.e., metallic nickel, could be detected, neither in the dark (resting state) nor under illumination (active state).

When the light is switched off, this structure decreases in intensity, thus confirming that the formation of Ni(I) is driven by illumination and is reversible. The fact that the Ni(I) feature at ca. 8337 eV disappears shortly after illumination indicates that such Ni(I) species are highly reactive, i.e., are short lived, according to

Ni^2+^+ e^–^ → Ni^+^_surface_ and 2Ni^+^_surface_ + 2H^+^ → H_2_ + 2Ni^2+^

The XANES spectra at the Ni K-edge of *white* TiO_2_ in a 0.4 mM solution of NiSO_4_ under different conditions are shown in Figure 4b. In the dark, the spectrum resembles that of Ni(II) hydrated ions, and under illumination, no difference can be detected. The same is valid for this sample in the dark after illumination. As shown in Figure 1, *white* TiO_2_ shows negligible photocatalytic activity, which is now further substantiated by the fact that no Ni(I) species form, neither in the dark nor under illumination, in the case of white titania.

The XANES spectra at the Ni K-edge of *black* TiO_2_ in a 0.4 mM solution of NiSO_4_ under different conditions are shown in Figure 4c. In the dark, the spectrum shows a spectral weight in the energy region of Ni(I), and this increases upon illumination. After illumination, the original spectral shape is recovered only after a long time in the dark (ca. 3 h). These conclusions are, however, made less straightforward by the fact that, in the energy region characteristic of N(I), also Ni(0) has an increased spectral weight. Nevertheless, these results seem to indicate that defective Ti^3+^-O_V_ sites in *black* TiO_2_ are highly active also in the dark, and a reaction with Ni(II) aqueous ions to form surface Ni(I) species takes place also without photogeneration of charge carriers in TiO_2_. Note that *black* TiO_2_, as shown in Figure 1, shows negligible photocatalytic activity.

**Figure 5:**
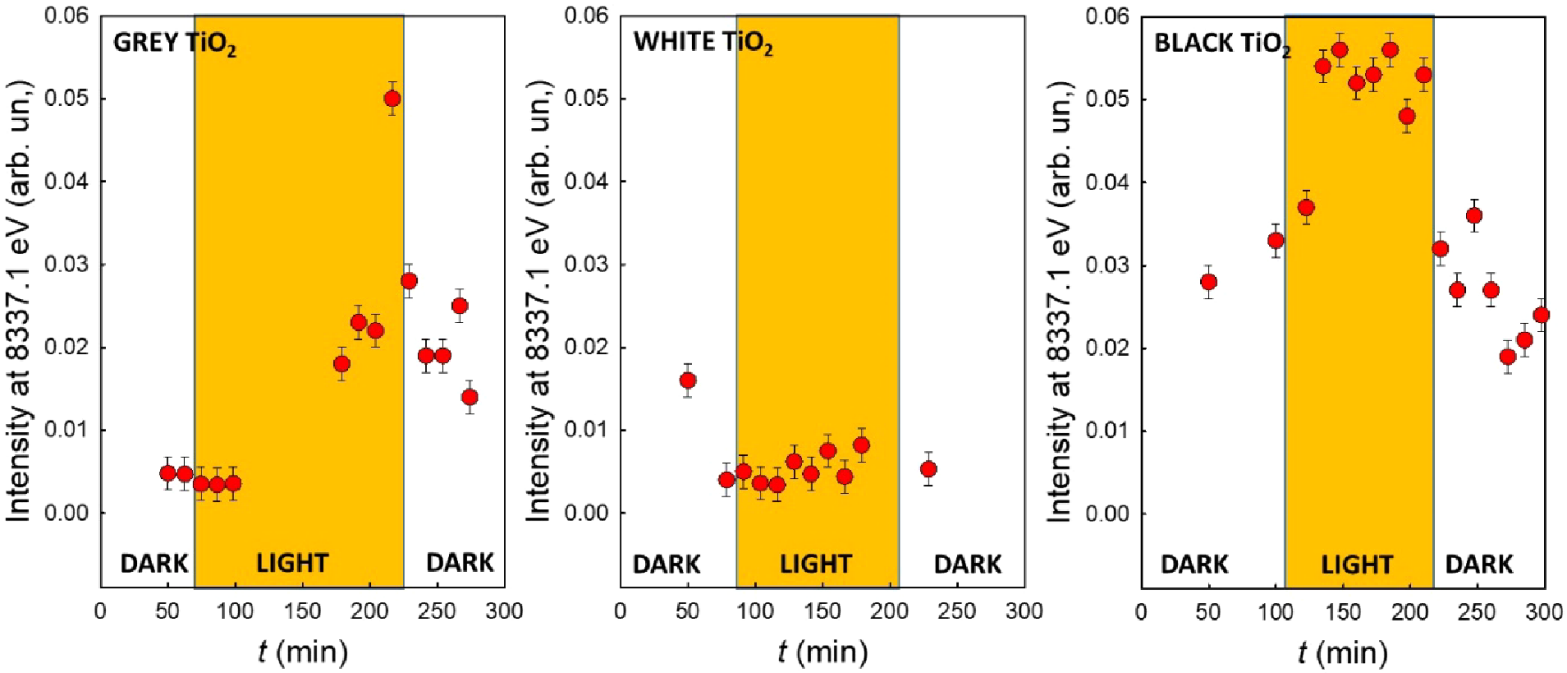
*In situ* time-resolved XAS data showing the trend of the absorption intensity at 8337.1 eV vs time for the different TiO_2_ samples.

*In situ* time-resolved XAS data help highlight the spectral differences between the three TiO_2_ samples, as shown in Figure 5. The value of the absorption intensity at 8337.1 eV, after normalization for the intensity at the main edge peak, is plotted as a function of time during the dark–light–dark cycles.

To sum up, the behavior of the three studied samples is very different. *White* TiO_2_ (center) is the simplest case: both under dark conditions and under UV illumination, the formation of Ni(I) can be excluded. For *gray* TiO_2_ (left) the formation of Ni(I) species requires UV illumination. The concentration of Ni(I) increases after an induction period, and decreases immediately when the illumination is switched off. The induction period resembles that observed during the photocatalytic H_2_ evolution experiments (see Figure 1). *Black* TiO_2_ (right), in contrast, shows the presence of Ni(I) species both in the dark and under illumination. Upon illumination, the concentration of Ni(I) increases only slightly and reaches soon a plateau. After switching off the illumination, it returns to its initial value.

Summarizing the results of photocatalytic H_2_ evolution experiments and *in situ* XAS, we observe that:

- Common to all parameter variations in photocatalytic H_2_ evolution experiments with *gray* TiO_2_ in aqueous NiSO_4_ solutions (Figures 1 and S2) is that, after starting UV illumination, an incubation period is observed until H_2_ is produced in detectable amounts. On the contrary, in the absence of Ni(II) salt, the H_2_ generation remains negligible.
- No Ni^0^, i.e., metallic nickel, can be detected, neither in the dark (resting state) nor under illumination (active state) for the tested photocatalyst.
- The XAS signal ascribed to Ni(I) species (Figure 5) increases over time under UV illumination, and so does the amount of generated H_2_ detected by GC analysis (Figures 1 and S2), suggesting that the two redox processes (Ni^+^ formation and H_2_ evolution) are linked, and likely the formation of Ni(I) species is the first redox step, followed by subsequent H_2_ generation.
- Results from our previous work^46^ suggest a strong correlation between the concentration of Ni(II) salt and the H_2_ generation (optimum of Ni(II) concentration 0.4 mM, as used in the present study). This can due to a trade-off between an increase in H_2_ generation activity, due to a higher concentration of photoformed Ni(I) states, and a decrease in effective reaction volume, due to a higher light absorbance of the aqueous phase with increasing the Ni(II) salt concentration.

Therefore, we conclude that

- Ni(I) species, formed on *gray* TiO_2_ but not on *white* TiO_2_, are key to catalyze the H_2_ evolution in the absence of a sacrificial agent and noble metal cocatalyst.
- The formation of Ni(I) species can be related to the presence of surface Ti^3+^ sites on TiO_2_, (confirmed by in situ EPR, below) which enable the formation of monovalent nickel (Ni(I)) species. The H_2_ evolution rate appears to correlate with the concentration of such surface Ni(I) species.
- To initiate the redox chain, i.e., the formation of Ni^+^ active species that enable H_2_ generation, needed are not only available Ni^2+^ ions in the solution but also Ti^3+^ sites at the TiO_2_ surface to react with Ni^2+^ ions. It is therefore expected that the highest H_2_ generation rates are achieved when the system is optimized in view of light absorption and availability of surface Ti^3+^ sites on TiO_2_ and Ni^2+^ ions in the reaction phase.
- Different hydrogenation treatments (different temperatures) form in TiO_2_ Ti^3+^-O_V_ sites of different density, distribution, and energy, in line with *in situ* EPR results discussed below and in our previous work.^70^
- Defective Ti^3+^-O_V_ structures in *black* TiO_2_ are highly reactive and form Ni(I) species already in the dark. This process, however, is not reversible, indicating that Ni(I) species formed on *black* anatase are stabilized by a strong interaction with the defective oxide surface and, hence, are less reactive. Hence, such Ni(I) species are significantly less reactive toward H_2_ evolution.

Note that *gray* titania provides not only an adequate surface density of Ti^3+^-O_V_ sites, but also a sufficient hole transfer ability to plain water, forming hydrogen peroxide, as shown in previous own work,^4^ in preliminary results in Figure S10, and discussed below along with *in situ* EPR results. This is crucial since, in the absence of efficient hole transfer, photoholes could oxidize the monovalent nickel (to Ni(II)), and this would impede the electron transfer to water for hydrogen evolution.

**Figure 6:**
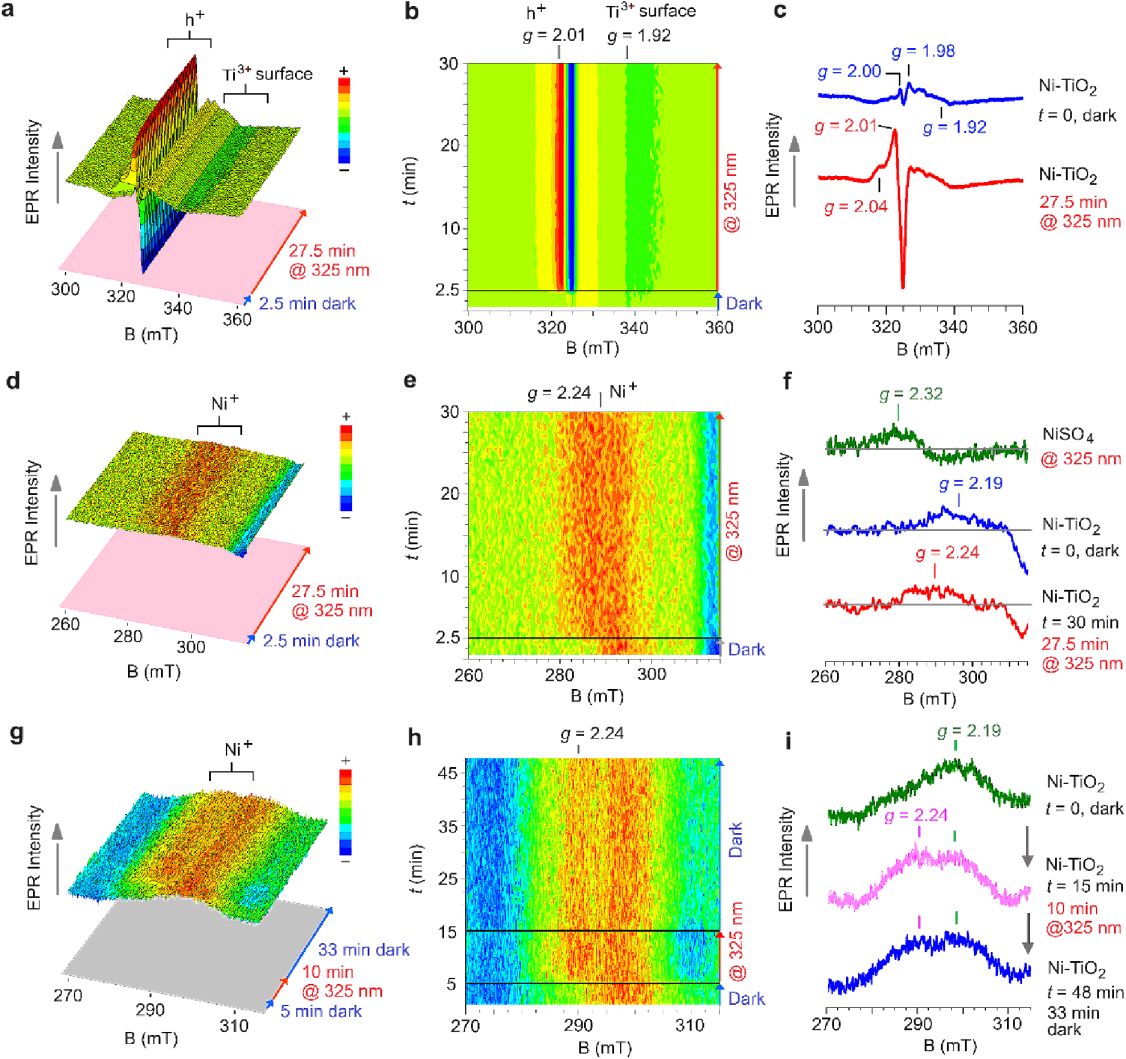
X-band (∼9.07–9.09 GHz) light-induced electron paramagnetic resonance (LEPR) spectra recorded at *T* = 80 K of *gray* TiO_2_ nanoparticles dispersed in an aqueous NiSO_4_ solution. Panels a–f: spectra were recorded in (oxygen containing) water, with and without light irradiation. Experimental conditions: X-band frequency 9.072 GHz, 0.4 mW applied power, 1.0 mT modulation width, 30 s sweep time for each sequential spectrum, and 0.03 s time constant. Panels g–i: spectra recorded in a 20 vol % MeOH/H_2_O mixture (nitrogen saturated), with and without light irradiation. Experimental conditions: X-band frequency: 9.086 GHz, 1.6 mW applied power, 0.6 mT modulation width, 60 s sweep time for each sequential spectrum, and 0.03 s time constant.

To validate our interpretation, we carried out *in situ* EPR experiments to investigate the effects of UV illumination on *gray* titania nanoparticles in Ni^2+^ aqueous solutions. Figure 6a (3D plot) and Figure 6d (2D plot) show *in situ* EPR resonance spectra (*T* = 80 K) of *gray* titania dispersed in a Ni^2+^ aqueous solution (in the absence of hole scavenger, e.g., MeOH). The sequential spectra were acquired under fast scanning conditions of the magnetic field (30 s) during a dark-light sequence (2.5 min dark followed by 27.5 min of irradiation at 325 nm, HeCd laser, power 200 mW). Under dark conditions, the recorded EPR spectra show a broad resonance signature developing in the 330–360 mT magnetic field range (*g*_avg_ ≈ 1.92, see Figure 6c, blue spectrum) and two resonance signals at lower magnetic field, at *g* = 1.98 and *g* = 2.00. These signals originate from Ti^3+^ states located in different lattice positions.^50,55^ The signals at *g* = 2.00 corresponds to electrons trapped into oxygen vacancies, Ti^3+^-O_V_, and the resonance at *g* = 1.98 corresponds to Ti^3+^ sites formed in bulk lattice positions. The broad signature at *g*_avg_ ≈ 1.92 originates from surface exposed Ti^3+^ sites.^50,54,55^ Weaker resonances are detected also in the lower magnetic field region (higher *g*-values), around *g* = 2.02–2.01; these resonances arise from trapped holes in the form of oxygen-based radicals (e.g., Ti–O^•^ species).

Soon after UV illumination, several changes become evident, as shown in the 3D (Figure 6a,d) and 2D plots (Figure 6b,e): (i) the appearance of the strong hole signature at *g*_1_ = 2.04 and *g*_2_ = 2.01 (see Figure 6c, red spectrum), (ii) the small increase of the signal of lattice embedded Ti^3+^ (*g*_avg_ = 1.92), best resolved in the 2D plot shown in Figure 6b, and importantly (iii) the formation of a new resonance feature at *g* ≈ 2.24 that can be assigned to the development of photoactive Ni^+^ sites (*S* = 1/2, 3d^8^4s^1^) (see Figure 6d,e).

The *g* ≈ 2.24 signal (see Figure 6f, red spectrum) is broad and very weak. This is due to the low concentration of Ni centers on the surface of *gray* titania along with different Ni coordination environments due to local disorder – this can explain the increase of the resonance line width. Note that before UV irradiation in the spectral range where the resonance at *g* ∼ 2.24 develops, another broad signal is also detected at *g* ∼ 2.19 (see the 2D plot in Figure 6e and the blue spectrum in Figure 6f). This resonance cannot arise from the presence of Ni^2+^ cations, because Ni^2+^ usually adopts the low spin state and is thus EPR silent (*S* = 0, 3d^8^). High spin state Ni^2+^, a non-Kamer system (*S* = 1), has such large zero-field-splitting that becomes undetectable at X-band frequencies.^65^ Ni^2+^ cations in water solution can be oxidized to Ni^3+^ giving an *S* = 1/2 configuration (3d^7^) upon illumination at 325 nm, which produces EPR spectral features (Figure S11, *g*_*z*_ = 2.32 and *g*_*x*_ = *g*_*y*_ = 2.06 that are different than those observed here with *gray* titania in the dark or upon illumination (see Figure 6f, blue and red spectra, respectivley). For example, a Ni^3+^-EDTA complex (*S* = 1/2) formed in aqueous solution upon irradiation of Ni^2+^-EDTA solutions was reported to exhibit EPR signatures at *g*_*z*_ = 2.336 and *g*_*x*_ = *g*_*y*_ = 2.139 (spectrum recorded at *T* = 77 K, ^60^Co γ-source).^66^ Therefore, the weak and broad resonance we observe under dark conditions at *g* ∼ 2.19 can be associated with the presence on the *gray* titania of a small fraction of less reactive Ni^+^ species that are strongly interacting with the defective oxide surface; such sites are already present before UV light irradiation, and form when the titania powder is suspended in the Ni salt solution. We propose that these Ni^+^ species form by electron transfer from surface exposed Ti^3+^ sites to Ni^2+^ cations. It should be noted that the concentration of such Ni^+^ species is rather low and just above the detection limit of EPR (a spin sensitive technique).

To gain further knowledge of the resonance signals developing in the *g* = 2.2 region and investigate the reversibility of photogeneration of Ni^+^ species, light-induced electron paramagnetic resonance (LEPR) experiments were performed at *T* = 80 K for the Ni^2+^-gray TiO_2_ water suspension upon addition of a small amount of MeOH as hole scavenger (20 vol % MeOH/H_2_O) and by using nitrogen saturated solutions. We applied a dark–light–dark sequence (5 min dark followed by 10 min of irradiation at 325 nm and then 33 min dark). Results are shown in Figure 6g (3D plot), Figure 6h (2D plot) and Figure 6i. In dark conditions at *t* = 0 min, the Ni^+^ signal at *g* = 2.19 (around 300 mT) becomes more resolved (Figure 6i, olive line), showing the presence of a shoulder at a lower magnetic field (275–290 mT). This shoulder, a distinct signal at *g* = 2.24 (see Figure 6g,h, time window 5–15 min, and Figure 6i, pink spectrum), belongs to highly photoactive Ni^+^ sites that are generated under UV irradiation of the sample in the EPR tube before freezing in liquid N_2_. When shutting down the UV light, the signal at *g* = 2.24 slowly decays at 80 K (see Figure 6g,h, time window 16–48 min, and Figure 6i, blue spectrum) while the signal at *g* = 2.19 does not. Therefore, we conclude that the signal at *g* = 2.24 belongs to the metastable Ni^+^ species, confirming the XANES results. We propose that, besides the fraction of Ni^+^ centers already formed in the dark in the as prepared materials (less reactive sites), surface adsorbed Ni^2+^ cations can act as (reversible) electron acceptors as soon as UV light is switched on, forming EPR active Ni^+^ species that are responsible for the photocatalytic H_2_ evolution activity of *gray* TiO_2_. The data point toward an electronic rearrangement that takes place between Ti^3+^ sites and adsorbed Ni^2+^ ions. Such reaction can be expressed in the form of: Ti^3+^ + Ni^2+^ → Ti^4+^–Ni^+^, and therefore the Ni^2+^ centers must be either located nearby a surface exposed Ti^3+^ site or trapped in a Ti^3+^–Ni^2+^ configuration.

**Figure 7:**
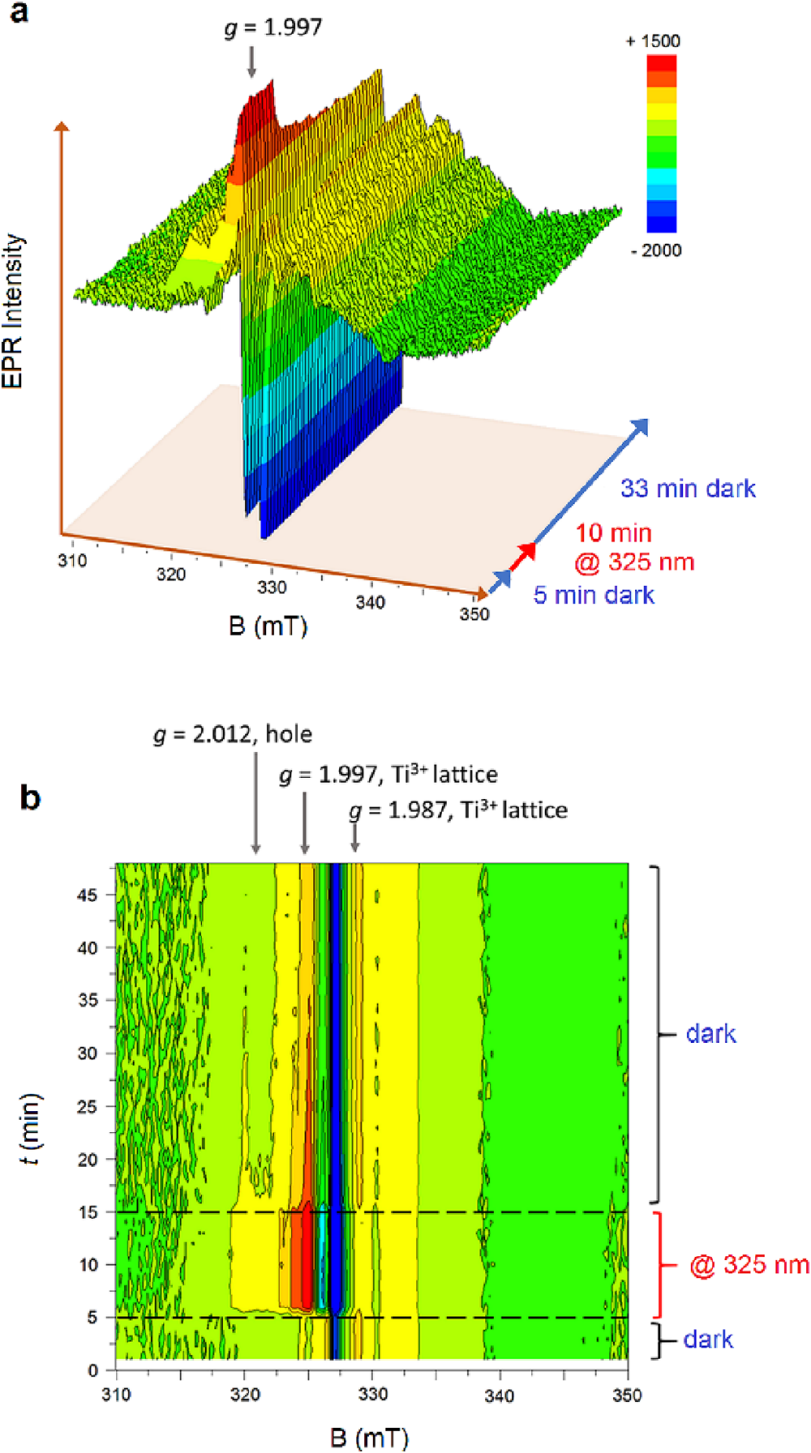
X-band LEPR spectra recorded at *T* = 80 K for *gray* TiO_2_ nanoparticles dispersed in NiSO_4_ solution (20 vol % MeOH/H_2_O, nitrogen saturated), with and without light irradiation. Sequence: 5 min dark, 10 min UV, and 33 min dark. Experimental conditions: X-band frequency: 9.085 GHz, 1.6 mW applied power, 0.6 mT modulation width, 60 s sweep time for each sequential spectrum, and 0.03 s time constant.

We also monitored by *in situ* LEPR experiments the magnetic field region where photogenerated holes and Ti^3+^ sites evolve (310–350 mT), using gray TiO_2_ in Ni–H_2_O/MeOH solutions. Spectra were recorded under identical conditions (5 min underthe dark followed by 10 min UV and then 33 min dark). Results are shown in Figure 7a,b (3D and 2D plots, respectively). Without the presence of dissolved oxygen, the spectra resolution is increased and allows to observe the formation of a new Ti resonance signature under light irradiation (*g* = 1.997, time 5–15 min) accompanied by the disappearance of the signal at *g* = 1.987 (Ti^3+^). The disappearance of this signal exactly correlates with the formation of Ni^+^ observed at *g* = 2.24.

Note that due to the presence of MeOH, the resonance associated with hole centers becomes much less dominant here, if compared to that observed under UV light in the absence of hole scavenger (shown in Figure 6a–c). The spectra in Figure 7a,b also show a small increment in the amount of surface exposed Ti^3+^ sites under UV light. As soon as the UV light is switched off (from *t* = 16 to 48 min), the signal at *g* = 2.012 slowly decreases (the hole centers), and so does the signal at *g* = 1.997. The signal at *g* = 1.987 (Ti^3+^) reforms while the excess of surface exposed Ti^3+^ sites generated under irradiation appears to slowly decrease over time.

By comparing the *in situ* EPR findings with the results of the photocatalytic experiments (Figure 1), and by considering the experimental differences between the photocatalytic and *in situ* EPR set-ups (temperature, illumination), we deduce that the activation toward H_2_ evolution occurs by a combination of a fast and a slow process. The fast process is the formation of metastable Ni^+^-gray titania complex, while the slow process is a reorganization of the Ti^3+^ defect centers.^46^ Hence, the ability of *gray* titania to evolve H_2_ in the absence of hole scavenger can be linked to the formation of Ni^+^-TiO_2_–Ti^3+^ sites; notably, and as shown in the *in situ* EPR spectra in Figure S12 (see inset), such active state is only metastable, i.e., when the sample is exposed to ambient conditions (room temperature, UV light switched off), the distribution of spin species formed under light (active state) relaxes back to the starting distribution seen under dark conditions (resting state), and H_2_ evolution ceases. In other words, the active form of the catalyst (Ni(I)-TiO_2-x_) is present as such only in operando, and it returns to a state close to the initial situation, i.e., inactive, once the light is switched off.

In previous work,^46^*in situ* EPR experiments showed that illumination of *gray* titania for several days in plain H_2_O in the absence of Ni^2+^ leads to a significant increase in the *g*_avg_ ≈ 1.93 signature, i.e., a strong increase in the amount of Ti^3+^ surface states occurs in the absence of Ni^2+^. This happens because swift electron transfer (to Ni cations in the solution) is not provided. In other words, TiO_2_ conduction band electrons are not transferred to the environment, and hence undergo trapping at Ti^3+^ surface states. In contrast, in the presence of Ni^2+^, even after 7 days of illumination, the intensity of the EPR signal at *g*_avg_ ≈ 1.92 remains almost unaltered. In other words, in the absence of Ni^2+^ in solution, under illumination, electron accumulation takes place, leading to a steady self-reduction of the *gray* anatase. In the presence of Ni^2+^, the electron transfer to the electrolyte (via the Ni^+^ surface relay) is sufficiently fast that self-reduction is limited.

**Figure 8:**
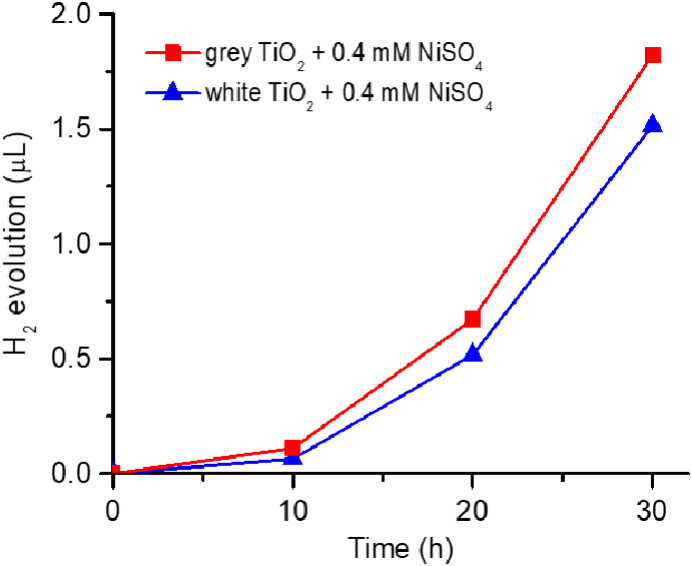
Comparison of H_2_ production rates of *gray* TiO_2_ nanoparticles vs *white* anatase TiO_2_ nanoparticles in a 0.4 mM NiSO_4_ methanol–water solution (50 vol % MeOH) under UV light irradiation (365 nm LED, 100 mW cm^–2^).

On the other hand, the important role of *gray* titania as hole transfer mediator^4^ is herein illustrated by comparing the behavior of *white* versus *gray* TiO_2_ in Ni^2+^-containing solutions in the absence or presence of methanol. In contrast to the inactive white form, *Gray* TiO_2_ in 0.4 mM NiSO_4_ aqueous solution not only evolves H_2_ (via  transfer to water) but also generates H_2_O_2_ after prolonged UV illumination (via  transfer to water, see H_2_O_2_ strip test in Figure S10). Instead, *white* TiO_2_ is found to evolve H_2_ from Ni^2+^-containing solutions only in the presence of MeOH (Figure 8); here, under illumination, methanol acts as the hole capturing agent, and hole-transfer is, thus, no longer the limiting factor. Note that in this case, H_2_ evolution takes place with *gray* and *white* TiO_2_ at comparable rate.

## Conclusions

In summary, in this work we demonstrated the light-induced self-assembly of a Ni^+^/TiO_2_/Ti^3+^ photocatalyst that is active for H_2_ generation from water in the absence of hole scavengers, e.g., methanol. This catalyst forms *in situ* when illuminating a suspension of reduced (*gray*) titania in a Ni^2+^ aqueous solution. No deposition of metallic Ni cocatalytic nanoparticles could be observed. Instead, we show that key to self-activation toward H_2_ evolution is the formation of monovalent Ni^+^ species on the TiO_2_ surface, combined with a light-induced rearrangement of defects in the semiconductor over time. Due to the metastable nature of the Ni^+^/TiO_2_/Ti^3+^ photocatalyst, *in situ* techniques (XAS and EPR) were used to prove the formation of monovalent Ni^+^ and the occurrence of defect reorganization. These self-assembly and activation processes enabling H_2_ evolution can be observed only for *gray* titania (and not for *white* or for *black* TiO_2_), as only *gray* titania provides surface Ti^3+^-O_V_ states with adequate energy to form active, metastable monovalent nickel species. *Gray* titania, in addition, provides the ability to transfer photoholes to plain water.

In a wider context, the present work demonstrates a self-assembly process where illumination is the synthesis tool for an active metastable entity, as well as the energy provider for this entity to achieve photocatalytic H_2_ evolution from water. Self-amplifying reaction schemes as observed in the present work may have considerable potential for simple one-pot synthesis and use of photocatalysts.

## Supporting Information Available

Methods; additional SEM, XRD, photocatalytic, XPS, cyclic voltammetry, XAS, H_2_O_2_ test strips, and EPR data; in situ XAS setup pictures; additional references (PDF)
