# Supplementary material for: Metastable Ni(I)-TiO2–x Photocatalysts: Self-Amplifying H2 Evolution from Plain Water without Noble Metal Co-Catalyst and Sacrificial Agent
Source: J Am Chem Soc. 2023 Nov 20;145(48):26122–32. doi: 10.1021/jacs.3c08199 (PMC10704555; doi:10.1021/jacs.3c08199)
Supplement: Supplementary file 1 — ja3c08199_si_001.pdf [file ja3c08199_si_001.pdf]

Supporting Information

A Metastable Ni(I)-TiO_{2-x} Photocatalyst: Self-Amplifying H_2 Evolution from Plain Water without Noble Metal Co-Catalyst and Sacrificial Agent

Marco Altomare[1], Shanshan Qin[2], Viktoriia A. Saveleva[3], Zdenek Badura[4,5], Ondrej Tomanec[4], Anca Mazare[2], Giorgio Zoppellaro[4,5], Alberto Vertova[6], Angelo Taglietti[7], Alessandro Minguzzi[6], Paolo Ghigna[7], and Patrik Schmuki[2,4]**

[1]PhotoCatalytic Synthesis PCS Group, MESA+ Institute for Nanotechnology, University of Twente, P.O. Box 217,7500 AE Enschede, The Netherlands

[2]Department Materials Science WW-4, LKO, Friedrich-Alexander-University of Erlangen-Nuremberg (FAU), 91058 Erlangen, Germany

[3]ESRF, The European Synchrotron, 71 Avenue des Martyrs, CS40220, 38043 Grenoble Cedex 9, France

[4]Regional Centre of Advanced Technologies and Materials, Czech Advanced Technology and Research Institute, Palacký University Olomouc, Křížkovského 511/8 779 00 Olomouc, Czech Republic

[5]Nanotechnology Centre, VŠB – Technical University of Ostrava, 17. listopadu 2172/15 708 00 Ostrava-Poruba, Czech Republic

[6]Dipartimento di Chimica, Università degli Studi di Milano, Via Golgi 19, 20133 Milan, Italy

[7]Dipartimento di Chimica, Università degli Studi di Pavia, Viale Taramelli 13, 27100 Pavia, Italy, Italy

* Corresponding authors. Email:

m.altomare@utwente.nl

schmuki@ww.uni-erlangen.de

Methods

Photocatalyst preparation. Anatase TiO_2 powder was purchased from Sigma Aldrich (99.7% purity, <25 nm particle size) and used as precursor of hydrogenated nanoparticles. As-purchased powder is referred to as *white* TiO_2. *Grey* TiO_2 nanoparticles were obtained through annealing in pure H_2 (Linde, 99.99%) at 500°C for 1 h in a tube furnace. To obtain different degrees of reduced TiO_2, the commercial anatase TiO_2 nanoparticles were annealed at 300, 400, 600 and 700°C in H_2. Particles treated for 1 h at 700°C are referred to as *black* TiO_2.[1] TiO_2 anatase sputtered layers were prepared through a method reported earlier.[2,3] *Grey* sputtered TiO_2 layer was obtained through annealing the as-sputtered TiO_2 layers in pure H_2 flow at 500°C for 1 h in a tube furnace.

SEM. A field-emission scanning electron microscope (FE-SEM) Hitachi FE-SEM 4800 was employed for morphology characterization.

XRD. For crystallographic characterization of the nanoparticles, X-ray diffraction analysis (XRD) was performed with an X'pert Philips MPD equipped with a Panalytical X celerator detector using graphite monochromized Cu Kα radiation (λ = 1.5406 Å).

XPS. X-ray photoelectron measurements (XPS, PHI 5600 XPS spectrometer, USA) were carried out for compositional information of the samples surface. An Al standard X-ray source with pass energy of 23.5 eV was used for XPS spectra. All XPS spectra were shifted to Ti 2p position of 458.5 eV.

TEM. A high-resolution transmission electron microscope (HR-TEM, FEI TITAN G2 60–300) was used for HAADF-STEM and EDS mapping of the samples.

Hydrogen evolution experiments. Photocatalytic H_2 generation experiments were conducted under open circuit conditions, employing a 365 nm LED (100 mW cm^{-2}) as a light source. 10 ml suspension containing 2 mg TiO_2 (*white*, *grey*, or *black* anatase nanoparticles) and 0.4 mM $NiSO_4{\cdot}6H_2O$ dissolved in deionized (DI) H_2O were placed in a quartz tube reactor sealed with a rubber septum. Before illumination, the suspension was sonicated for 10 minutes and purged with Ar for 20 minutes to remove O_2 from the solution and head space in the quartz tube reactor. A gas chromatograph (GCMS-QO2010SE, Shimadzu) (GC) with thermal conductivity detector was used to determine the amount of H_2 generated. The sample was continuously stirred during irradiation and GC measurements were conducted to evaluate the amount of generated H_2 over illumination time. The same procedure for the hydrogen evolution test was conducted for *grey* TiO_2 nanoparticles in 0.004 mM, 0.04 mM, 4 mM and 40 mM $NiSO_4{\cdot}6H_2O$ aqueous solutions (prepared from deionized H_2O). In a control experiment for hydrogen evolution, 2 mg TiO_2 (*white* or *grey* anatase nanoparticles) in 10 ml of 0.4 mM $NiSO_4{\cdot}6H_2O$ dissolved in H_2O:methanol solution (50:50 vol.%) were illuminated with a 365 nm LED (100 mW/cm^2). A Brinell funnel was used to collect Ni-grey TiO_2 powders after UV illumination. The entire drying and collection process was conducted in an Ar gas filled glove box.

Synthesis of $[Ni(cyclam)]^{2+}$. $[Ni(cyclam)]^{2+}$ (cyclam = 1,4,8,11-tetraazacyclotetradecane) complex was prepared as a perchlorate salt, $[Ni(cyclam)](ClO_4)_2$, following a method reported in the literature.[4]

Electrochemical experiments. Electrochemical experiments were conducted in a solution containing 5 mM $[Ni(cyclam)](ClO_4)_2$ and 0.1 M tetrabutylammonium tetrafluoroborate in

acetonitrile, previously degassed with N_2 for 10 minutes. A Pt wire and an Ag wire were used as the counter and a pseudoreference electrode, respectively. A description of the cell can be found in our recent papers on electrochemical and photo-electrochemistry XAS.[5,6] Briefly, it is made of a silicon rubber structure that holds two plastic walls transparent to visible light. The window in front of the working electrodes is made of thin Mylar® foil to guarantee a negligible X-ray absorption.

In-situ XAS. The experiments were performed at the beamline ID26 of the European Synchrotron Radiation Facility (ESRF), Grenoble, France. The storage ring operated in 7/8 multibunch mode with an electron current of 200 mA. Three undulators produced the incoming radiation, which was monochromatized by a pair of Si (311) crystals, cryogenically cooled. The energy calibration of the incident beam was done using a reference metallic nickel foil by setting the first reflection point of Ni K edge to 8333 eV. Ni Kα high energy resolution fluorescence detected (HERFD) X-ray absorption near edge structure (XANES) spectra were collected in a continuous scan mode. The maximum of the Ni Kα emission line (7478.2 eV) was selected using 620 reflection of five Ge crystals in Rowland geometry (Bragg angle of 67.93°). Si avalanche photodiode (APD) with 200 μm thickness and 10 x 10 mm^2 active area was used as a detector.
The experiments were performed on aqueous TiO_2 photocatalyst suspensions in the presence of Ni(II) ions under simulated solar light illumination. Our aim was to confirm the formation of Ni(I) species and the dependence of such self-assembly process on the defective structure (Ti^{3+}-O_V) of differently reduced titania powders. We performed the measurements in aqueous suspensions in the presence of $NiSO_4$, with or without UV illumination, and recorded XANES spectra at the Ni-K edge. In each experiment, 30 mg of photocatalyst were dispersed in 3 mL of a 0.4 mM $NiSO_4$ aqueous solution. The suspensions were sonicated for 10 minutes and then loaded into the in-situ XAS cell. Each experiment was followed for up to 5 h, to track the formation kinetic of Ni(I) species, alternating dark and illumination periods, each of some 2 h (i.e., intermittent illumination). We used an in-situ XAS photocatalytic cell (see Fig. S7) already adopted in previous work.[7] UV illumination to activate the photocatalyst was provided by a 365 nm LED (power density 185 mW cm^{-2}). The TiO_2 photocatalyst slurry was kept under vigorous stirring to avoid sedimentation. The entire volume of photocatalyst slurry was illuminated. This was crucial to ensure that the signal acquired originated from particles exposed to the same (homogeneous) experimental conditions. The in-situ XAS cell had a Mylar® window to grant transparency for (incoming and fluorescence) X-rays and UV light. We used Ar gas to purge the reaction phase (to remove dissolved O_2).
Fig. 5 in the main text reports the absorption coefficients at 8337.1 eV in the pertinent XAS spectra at the Ni K-edge. Due to noise, for obtaining these values, we averaged the experimental absorption in a relevant spectral range, i.e., in a 4 eV-wide range centered around 8337.1 eV. The error of each value is given as the standard deviation of the absorption values in the interval, and the error bars in Fig. 5 refer to these standard deviations.

In-situ EPR. X-band Electron paramagnetic resonance (EPR) spectra were recorded at the temperature of 80 K and collected on a JEOL JES-X-320 spectrometer equipped with variable temperature control ES 13060DVT5 apparatus. The cavity Q quality factor was kept above 6000 in all measurements. Highly pure quartz tubes were employed (Suprasil, Wilmad, ⩽ 0.5 OD) and accuracy on *g*-values was obtained against a Mn^{2+}/MgO standard (JEOL standard). The microwave power was set to be lower than < 2.00 mW, to avoid saturation effects. For the *in-situ* experiments, a HeCd laser operating at 325 nm was used (200 mW) by direct fitting through optical wire the light source into the dedicated optical window of the resonator.

Additional results

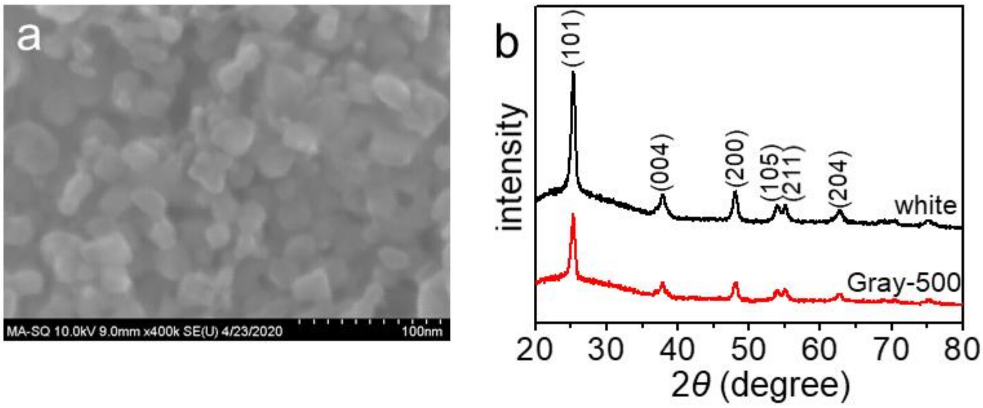

Fig. S1. a) SEM image of *grey* anatase nanoparticles. b) X-ray diffraction patterns for commercial *white* nanoparticles and the *grey* nanoparticles.

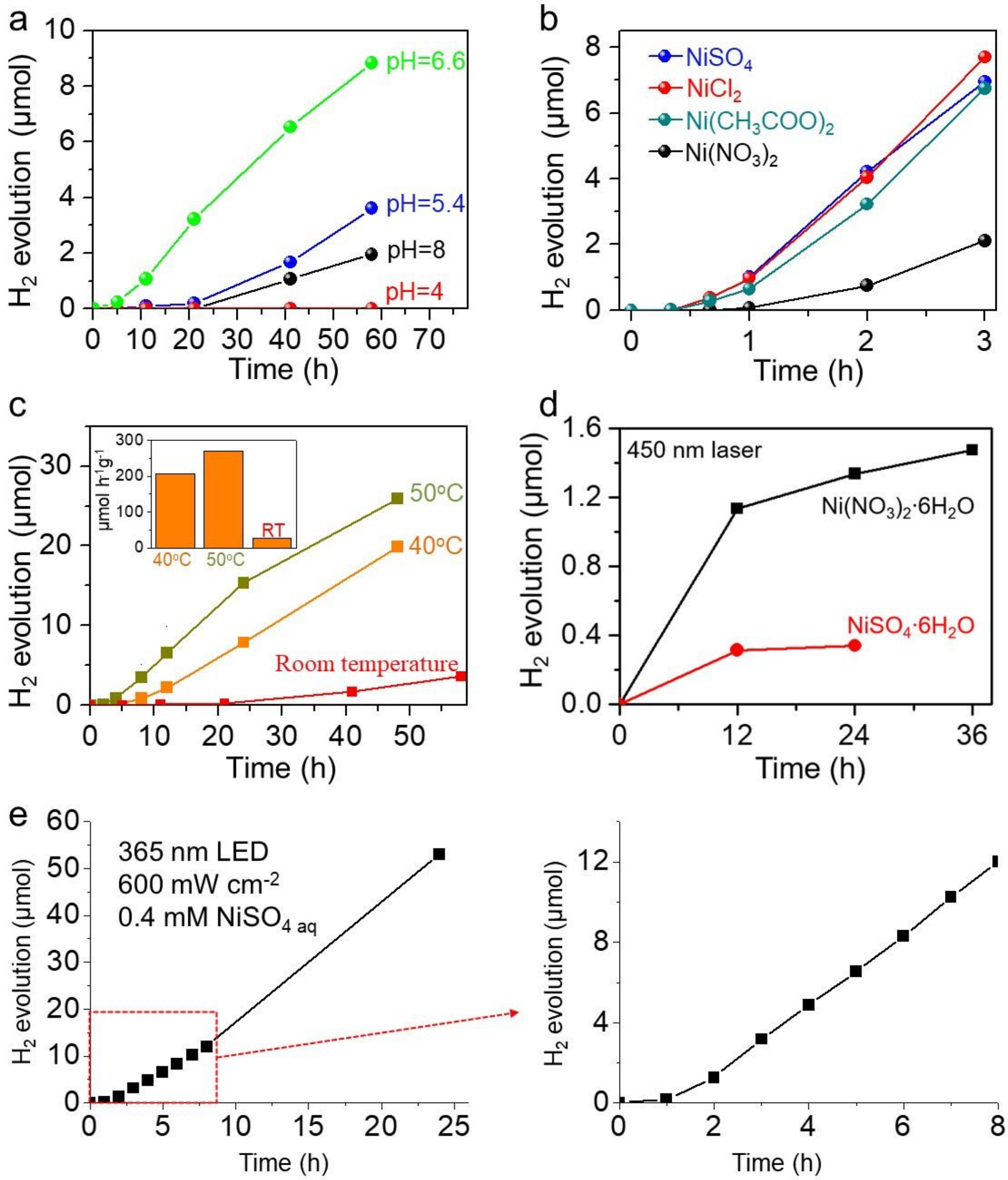

Fig. S2. Effect of a) pH, b) Ni salt precursor and c) temperature on the photocatalytic H_2 evolution rate. Conditions: *Grey* anatase nanoparticles, 0.4 mM Ni^{2+} aqueous solution, illumination by 365 nm LED (100 mW cm^{-2}). d) H_2 generation of *grey* TiO_2 measured under illumination with a 365 nm LED (100 mW cm^{-2}) for 12 h in 0.4 mM $Ni(NO_3)_2{\cdot}6H_2O$ or 0.4 mM $NiSO_4{\cdot}6H_2O$ in H_2O:methanol=50:50 vol.% solution, followed by Ar purging for 25 min (to completely remove H_2) and then by illumination with a 450 nm laser (2 W). e) Effect of light power density on the photocatalytic H_2 evolution rate. Conditions: *Grey* anatase nanoparticles, 0.4 mM Ni^{2+} aqueous solution, illumination by 365 nm LED, 600 mW cm^{-2}.

Fig. S2d shows that the H_2 generation photo-chemistry can be driven by visible light. This is achieved by illumination of *grey* TiO_2 with a 365 nm LED (100 mW cm^{-2}) for 12 h in 0.4 mM $Ni(NO_3)_2{\cdot}6H_2O$ or 0.4 mM $NiSO_4{\cdot}6H_2O$ in H_2O:methanol=50:50 vol.% solution, followed by

Ar purging for 25 min (to completely remove H_2), and then by illumination with a 450 nm laser (2 W). The pre-illumination with UV light leads to self-reduction of titania, due to Ti^{3+}−O_v formation, and these states are then activate for photocatalytic hydrogen generation under visible light.

Fig. S2e shows the results of testing *grey* TiO_2 for H_2 evolution under a higher power intensity, i.e., 600 mW cm^{-2}, 365 nm LED. The data show a reduced induction time compared to the experiments at 100 mW cm^{-2} (Fig. 1, main text), and a much higher amount of generated H_2 after a certain time, i.e., 55 vs. 0.3 μmol_{H_2} after 24 h, this by using a 6 times higher UV illumination power.

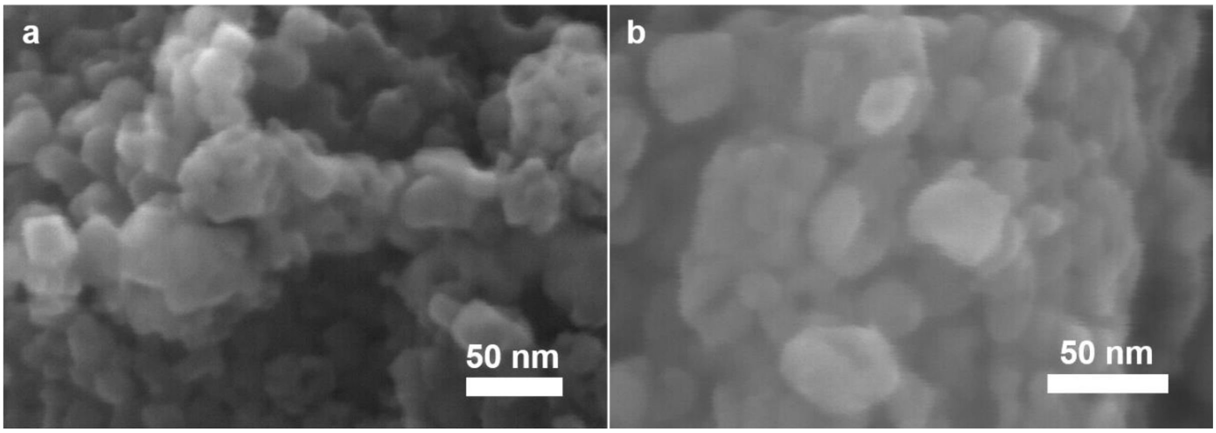

Fig. S3. SEM images of *grey* anatase nanoparticles in 0.4 mM of $NiSO_4{\cdot}6H_2O$ aqueous solution after 10 days of illumination by 365 nm LED (100 mW/cm^2).

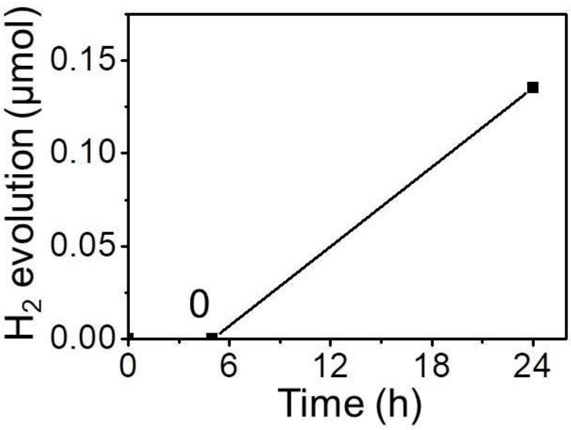

Fig. S4. H_2 evolution performance of *grey* sputtered TiO_2 layer illuminated by 365 nm LED (100 mW/cm^2).

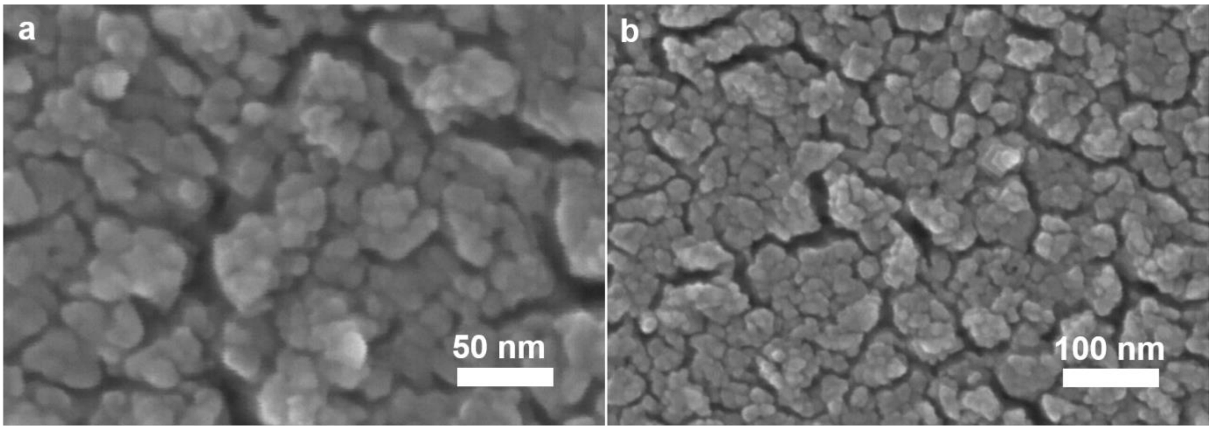

Fig. S5. SEM images of *grey* anatase sputtered TiO_2 layer in 0.4 mM of $NiSO_4{\cdot}6H_2O$ aqueous solution after 24 h of illumination by 365 nm LED (100 mW/cm^2).

Table S1. Surface atomic concentration (at. %) from XPS data

**Carbon**	**Oxygen**	**Titanium**	**Nickel**
33.96	53.98	12.55	0.51

The XPS-derived atomic concentration data in Table S1 are for a polycrystalline TiO_2 anatase layer, produced by magnetron sputtering (on SiO_2/Si), reduced (hydrogenation at 500°C, 1 h), and exposed for 24 h to UV light (365 nm), in a 0.4 mM $NiSO_4 \cdot 6H_2O$ aqueous solution. The data reveal a Ni surface content of ca. 0.5 at%, and a Ni:Ti ratio of ca. 0.04:1, indicating a relatively low loading of Ni species.

Table S2. Peak fit parameters for the Ni2$p_{3/2}$ peak and the corresponding satellite in Fig. 2e (main text).

**$Ni^{\delta+}$ (72.4%)**					
	**$^{\delta+}$-1**	**$^{\delta+}$-2**	**$^{\delta+}$-3**	**$^{\delta+}$-4**	**$^{\delta+}$-5**
**Binding energy, eV**	853.58	855.78	861.28	864.58	867.08
**FWHM**	3.50	2.60	3.05	4.00	4.5
**%Area**	7.35	18.39	14.12	4.41	3.31
**Ni^{2+} (27.6%)**					
	**$^{2+}$-1**	**$^{2+}$-2**	**$^{2+}$-3**	**$^{2+}$-4**	**$^{2+}$-5**
**Binding energy, eV**	856.41	858.51	864.01	867.11	869.49
**FWHM**	2.83	2.98	2.98	3.32	3.78
**%Area**	2.13	7.78	4.16	0.93	1.21

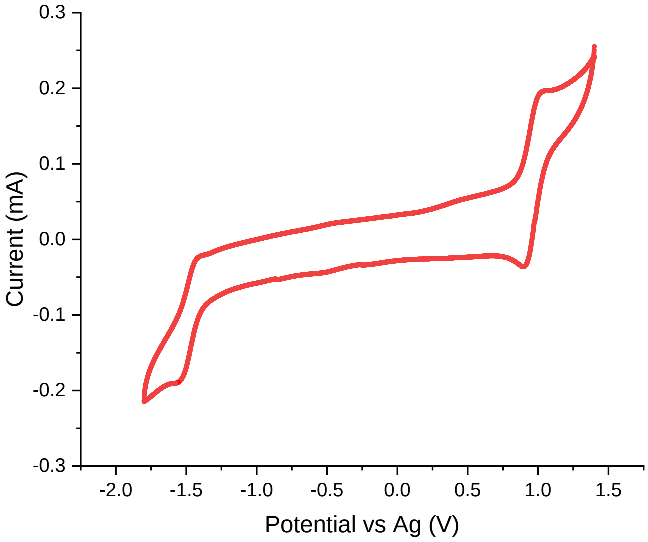

Fig. S6. Cyclic voltammetry of [Ni(cyclam)](ClO_4)$_2$ in acetonitrile at 20 mV s^{-1}. The cyclic voltammetry shows two sets of peaks related to reversible processes. These are due either to the Ni(III)/Ni(II) couple at about 1.1 V or the Ni(II)/Ni(I) one at about -1.4 V.[8]

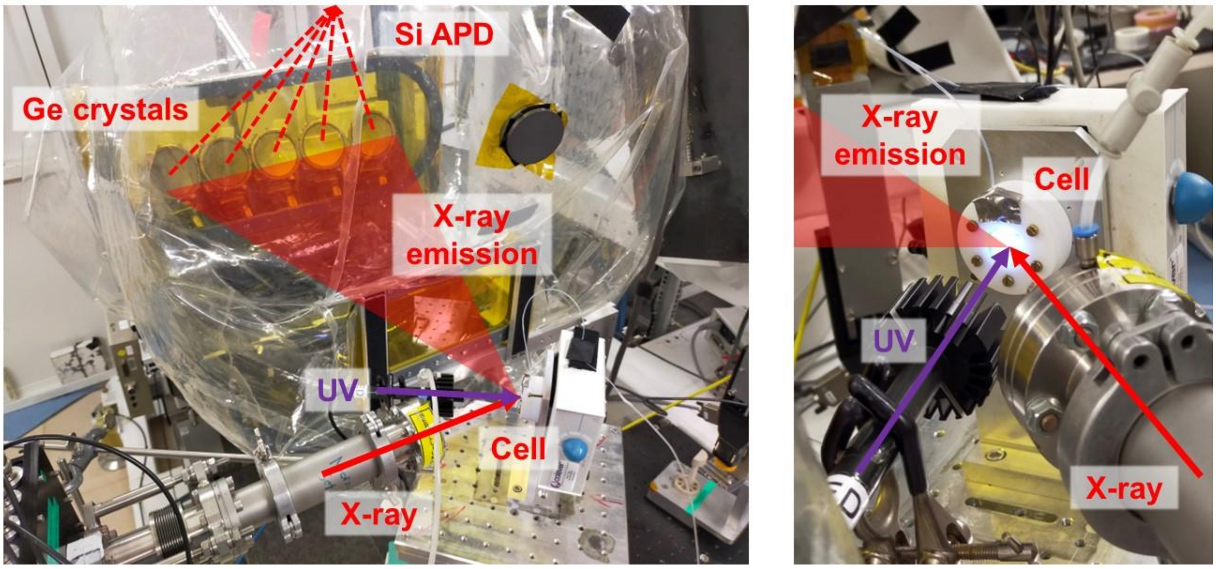

Fig. S7. *In-situ* XAS setup at ID26 (ESRF).

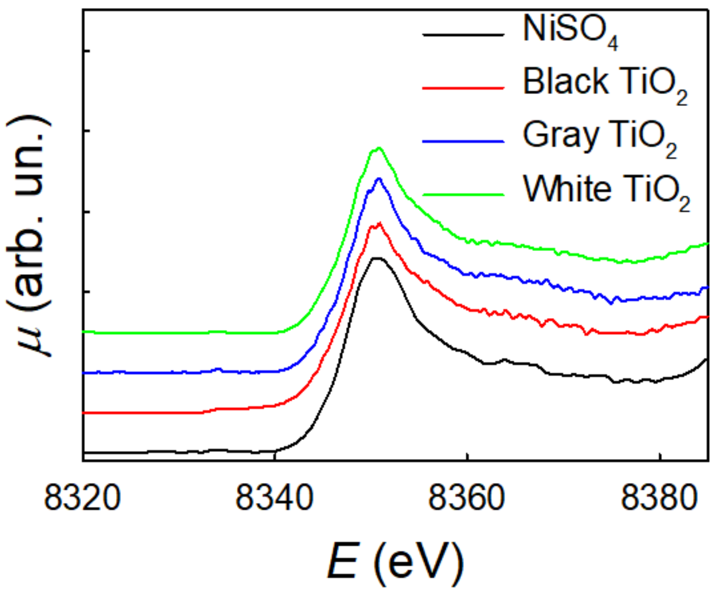

Fig. S8. Ni K-edge XANES spectra of a 0.4 mM solution of $NiSO_4$ (black line), and of the same solution in the presence of *black* (red), *grey* (blue), and *white* (green) TiO_2 nanoparticles. All spectra refer to dark conditions.

Fig. S8 shows the Ni K-edge XANES spectrum of a 0.4 mM solution of $NiSO_4$, compared to the spectra of the same solution in the presence of black, grey, and white TiO_2, all in dark conditions. The close similarity of the edge energy position for all the spectra confirms that the oxidation state is Ni(II) in every condition. Minor differences are visible when the solution is put in contact with the different TiO_2 nanoparticles, which may be indicative of the formation of different Ni(II) species adsorbed onto the TiO_2 nanoparticles.

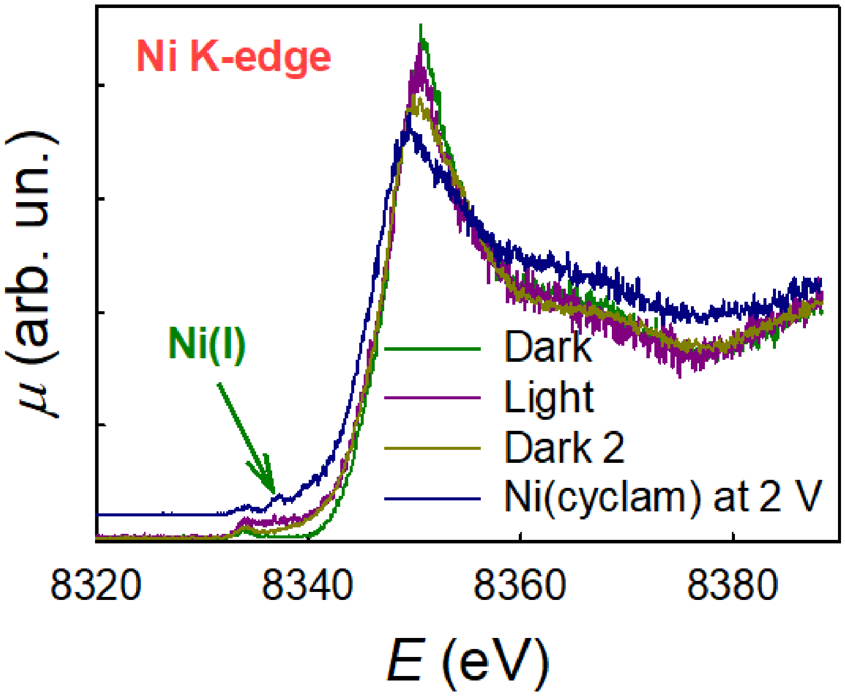

Fig. S9. Ni K-edge XANES of Ni(I) from **[Ni(cyclam)]$^{2+}$**overlapped with the XANES spectra of the *grey* TiO_2 samples in different conditions. The spectra of **[Ni(cyclam)]$^{2+}$** is shifted on the *y* axis for better clarity.

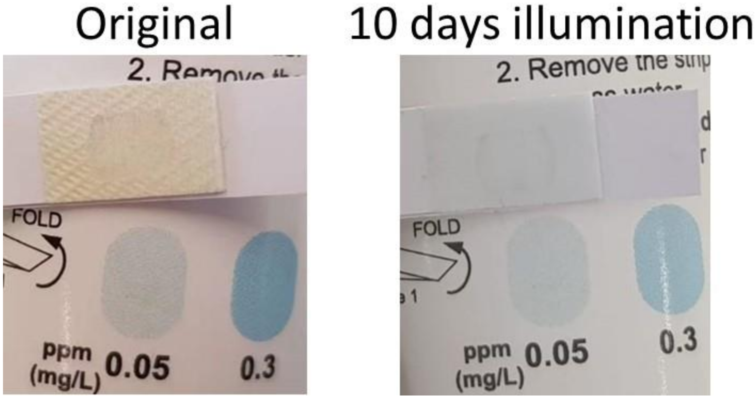

Fig. S10. H_2O_2 test strips showing the formation of H_2O_2 after prolonged UV illumination of *grey* TiO_2 in a 0.4 mM $NiSO_4{\cdot}6H_2O$ aqueous solution.

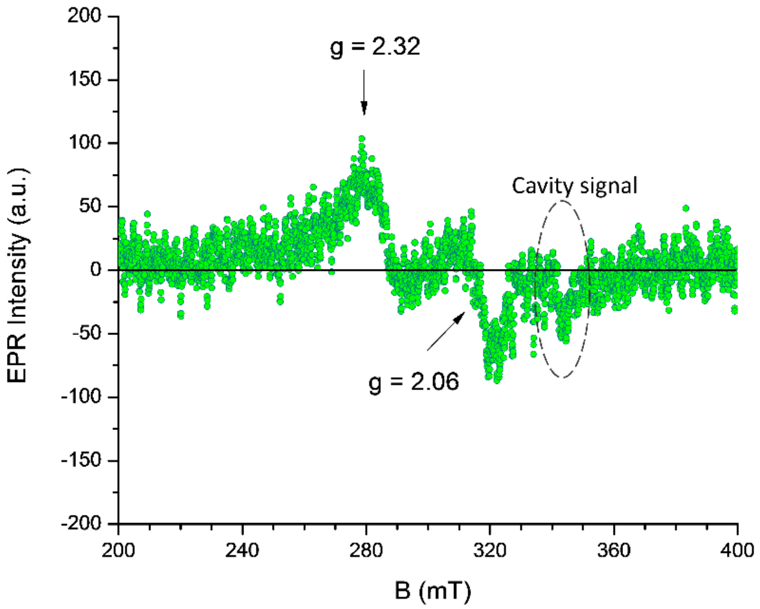

Fig. S11. EPR spectrum of Ni(II)$(SO_4)_2$ in water recorded in frozen matrix at 78 K upon UV light illumination (325nm, 20 min). Experimental conditions: Frequency 9.086 GHz, 1.6 mW applied power, 0.6 modulation width, 0.03 s time constant, 4 min sweep time.

The spectra in Fig. S11 highlights that a small percentage of Ni(II) in water (exposed to ambient air) is oxidized to Ni(III).

The observed g-values for Ni^{3+} and Ni^{+} species not only depends on the Ni oxidation state, i.e., +3 or +1 (assuming for both S=1/2), but also on the ligand-field in which the Ni cation is embedded, as supported by the following examples from the literature.

- Ni(III)[9] complexes obtained by in-situ oxidation of Ni(II) with magic blue and cerium ammonium nitrate can give EPR signatures for Ni(III) with large g-tensor values, as large as g=2.32, 2.23, 2.01.
- The Ni(III) cation in NiSOD[10] exhibits EPR spectrum displaying an S=1/2 signal at g_x=2.30, g_y=2.24, and g_z=2.01.
- In NiFe Hydrogenase,[11] the measured g-values for Ni(III) are 2.33, 2.16 for Ni-B and 2.32, 2.24 for Ni-A.

It is crucial to consider the EPR signals observed for the aqueous Ni^{2+} solution exposed to air and under light irradiation (Fig. S11) vs. the behaviour observed for the Ni/TiO_2 system under light irradiation, which gives very different EPR features.

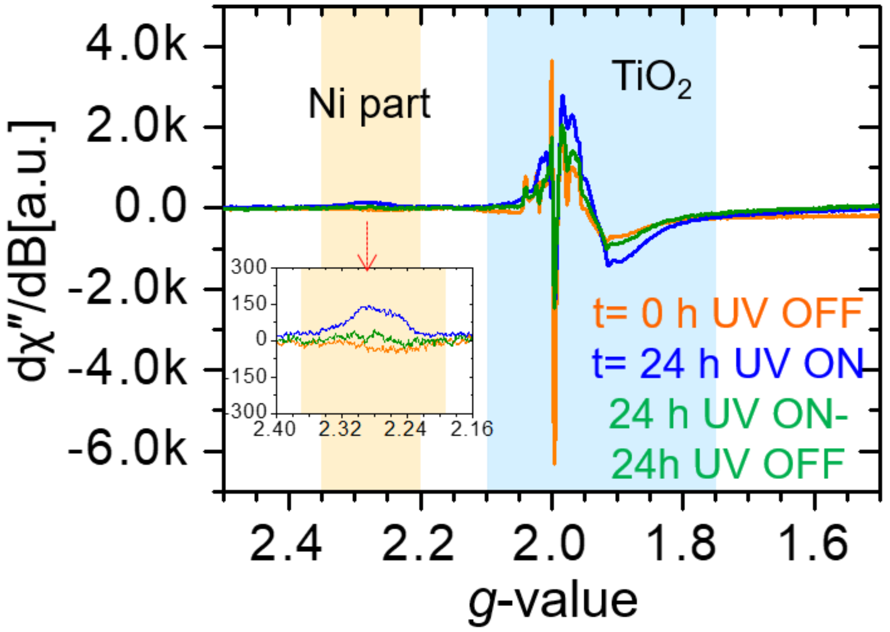

Fig. S12. X-band (~9.14–9.17 GHz) EPR spectra recorded at T=78 K of *grey* TiO_2 nanoparticles (hydrogenated at 500°C) dispersed in $NiSO_4 \cdot 6H_2O$ solution.

The inset highlights the Ni part of the EPR signal. The orange line shows the spectrum of a fresh sample recorded without light exposure. The blue trace was recorded after 24 h of 325 nm laser irradiation (200 mW). Then, the sample was resting in dark for 24 hours and the green spectrum was recorded (measured without UV irradiation). This further illustrates the metastable nature of the catalyst species.

References

(1) Liu, N.; Zhou, X.; Nguyen, N. T.; Peters, K.; Zoller, F.; Hwang, I.; Schneider, C.; Miehlich, M. E.; Freitag, D.; Meyer, K.; et al. Black Magic in Gray Titania: Noble-Metal-Free Photocatalytic H_2 Evolution from Hydrogenated Anatase. *ChemSusChem* **2017**, *10*, 62-67.

(2) Kim, H.; Wang, Y.; Denisov, N.; Wu, Z.; Kment, Š.; Schmuki, P. DC sputter deposited TiO2 layers on FTO: towards a maximum photoelectrochemical response of photoanodes. *J. Mater. Sci.* **2022**, *57*, 12960-12970.

(3) Hejazi, S.; Mohajernia, S.; Osuagwu, B.; Zoppellaro, G.; Andryskova, P.; Tomanec, O.; Kment, S.; Zbořil, R.; Schmuki, P. On the Controlled Loading of Single Platinum Atoms as a Co-Catalyst on TiO2 Anatase for Optimized Photocatalytic H2 Generation. *Adv. Mater.* **2020**, *32*, 1908505.

(4) Barefield, E.; Wagner, F.; Herlinger, A.; Dahl, A. In Inorganic Synthesis; Basolo, F., Ed. McGraw-Hill: New York: 1976; Vol. 16, p 220.

(5) Fracchia, M.; Cristino, V.; Vertova, A.; Rondinini, S.; Caramori, S.; Ghigna, P.; Minguzzi, A. Operando X-ray absorption spectroscopy of WO3 photoanodes. *Electrochim. Acta* **2019**, *320*, 134561.

(6) Malara, F.; Fracchia, M.; Kmentová, H.; Psaro, R.; Vertova, A.; Oliveira de Souza, D.; Aquilanti,G.; Olivi, L.; Ghigna, P.; Minguzzi, A.; et al. Direct Observation of Photoinduced Higher Oxidation States at a Semiconductor/Electrocatalyst Junction. *ACS Catal.* **2020**, *10*, 10476-10487.

(7) Spanu, D.; Minguzzi, A.; Recchia, S.; Shahvardanfard, F.; Tomanec, O.; Zboril, R.; Schmuki, P.; Ghigna, P.; Altomare, M. An Operando X-ray Absorption Spectroscopy Study of a NiCu−TiO_2 Photocatalyst for H_2 Evolution. *ACS Catal.* **2020**, *10*, 8293-8302.

(8) Lovecchio, F. V.; Gore, E. S.; Busch, D. H. Oxidation and reduction behavior of macrocyclic complexes of nickel. Electrochemical and electron spin resonance studies. *J. Am. Chem. Soc.* **1974**, *96*, 3109-3118.

(9) Pirovano, P.; Twamley, B.; McDonald, A. R. Modulation of Nickel Pyridinedicarboxamidate Complexes to Explore the Properties of High-valent Oxidants. *Chemistry – A European Journal* **2018**, *24*, 5238-5245.

(10) Choudhury, S. B.; Lee, J.-W.; Davidson, G.; Yim, Y.-I.; Bose, K.; Sharma, M. L.; Kang, S.-O.; Cabelli, D. E.; Maroney, M. J. Examination of the Nickel Site Structure and Reaction Mechanism in Streptomyces seoulensis Superoxide Dismutase. *Biochemistry* **1999**, *38*, 3744-3752.

(11) Geßner, C.; Trofanchuk, O.; Kawagoe, K.; Higuchi, Y.; Yasuoka, N.; Lubitz, W. Single crystal EPR study of the Ni center of NiFe hydrogenase. *Chemical Physics Letters* **1996**, *256*, 518-524.
